# Immune Milieu Established by Postpartum Liver Involution Promotes Breast Cancer Liver Metastasis

**DOI:** 10.3390/cancers13071698

**Published:** 2021-04-03

**Authors:** Alexandra Q. Bartlett, Nathan D. Pennock, Alex Klug, Pepper Schedin

**Affiliations:** 1 Department of Cell, Developmental, and Cancer Biology, Oregon Health & Science University, Portland, OR 97239, USA; quackena@ohsu.edu (A.Q.B.); pennock@ohsu.edu (N.D.P.); klug@OHSU.edu (A.K.); 2 Knight Cancer Institute, Oregon Health & Science University, Portland, OR 97201, USA; 3 Young Women’s Breast Cancer Translational Program, University of Colorado Anschutz Medical Campus, Aurora, CO 80045, USA

**Keywords:** metastatic niche, liver metastasis, breast cancer, liver involution

## Abstract

**Simple Summary:**

Cancer becomes lethal when it metastasizes to secondary sites, and for breast cancer metastasis to the liver is a serious clinical problem. Liver metastasis is promoted, in part, by changes to the liver environment, resulting in the formation of a metastatic niche that supports circulating tumor cells. Understanding how the liver niche support breast cancer cells may lead to development of treatments for patients with metastatic breast cancer. Here, we report that the developmentally regulated process of weaning-induced liver involution increases liver metastasis in cancer cells with otherwise low metastatic potential. Increased metastasis associates with unique immunological properties in the involuting liver, including reduced ability to activate T cells required for tumor cell clearance. These data establish physiologic liver involution as a model to understand the liver metastatic niche and suggest future research into whether the immune milieu identified in the involuting liver could be targeted to treat metastases more generally.

**Abstract:**

In rodents, we identified a physiologic process within the normal liver that creates a pre-metastatic niche. This physiology is weaning-induced liver involution, characterized by hepatocyte cell death, immune influx, and extracellular matrix remodeling. Here, using weaning-induced liver involution as a model of a physiologically regulated pro-metastatic niche, we investigate how liver involution supports breast cancer metastasis. Liver metastases were induced in BALB/c immune competent hosts by portal vein injection of D2OR (low metastatic) or D2A1 (high metastatic) mouse mammary tumor cells. Tumor incidence and multiplicity increased in involution hosts with no evidence of a proliferation advantage. D2OR tumor cell extravasation, seeding, and early survival were not enhanced in the involuting group compared to the nulliparous group. Rather, the involution metastatic advantage was observed at 14 days post tumor cell injection. This metastatic advantage associated with induction of immune tolerance in the involution host liver, reproductive state dependent intra-tumoral immune composition, and CD8-dependent suppression of metastases in nulliparous hosts. Our findings suggest that the normal postpartum liver is in an immune suppressed state, which can provide a pro-metastatic advantage to circulating breast cancer cells. Potential relevance to women is suggested as a postpartum diagnosis of breast cancer is an independent predictor of liver metastasis.

## 1. Introduction

Breast cancer deaths are almost exclusively due to spread of the cancer to distant organs, i.e., metastasis, and young women with breast cancer are at a higher risk of dying from metastasis than older women [1,2]. The dominant paradigm to explain the increased deaths in younger women is that their tumors have intrinsic properties that increase metastatic capability. In support of this hypothesis, young women are at increased risk of being diagnosed with poor prognostic HER2+ and triple negative (ER-PR-HER2-) breast cancers [2,3]. However, research shows that young breast cancer patients have worse outcomes compared to older peers regardless of cancer subtype [4]. This suggests that tumor extrinsic factors, such as the microenvironment at the secondary site, might also contribute to increased metastasis observed in young women. Recently, efforts to investigate secondary environments permissive of metastatic growth have gained momentum as an approach to better understand and target metastasis [5,6,7,8].

Sites within secondary organs that support tumor cell survival and proliferation are called “metastatic niches” [6]. Stages of the metastatic process that the niche support include tumor cell adherence to organ-specific endothelium, extravasation, survival, immune avoidance, and outgrowth [9]. These pro-metastatic effects are mediated by niche components including insoluble extracellular matrix (ECM) proteins, soluble proteins, and stromal cells such as fibroblasts, endothelial cells, and immune cells. The evolution of an organ into a pro-metastatic niche has been attributed to tumor education, whereby a primary tumor exerts systemic effects that remodel the distant site to a more permissive state [7,10,11,12,13,14]. However, a largely unexplored hypothesis that may further our understanding of breast cancer metastasis in young women is the idea that physiologic remodeling of secondary sites can form a pro-metastatic niche. To date, how normal physiology impacts a possible metastatic niche is not well-studied, in part due to lack of suitable model systems.

One example of a normal, physiologic process that creates a pro-metastatic niche is weaning-induced liver involution. In rodents, it has been reported that the liver approximately doubles in size during pregnancy and retains elevated size throughout lactation, putatively to accommodate the increased metabolic demands of pregnancy and lactation [15,16,17,18]. Liver involution returns the pregnancy and lactation-enlarged liver to a pre-pregnant state via a process that includes hepatocyte programmed cell death, catabolic metabolism, ECM remodeling, and immune cell influx [18]. Of note, during liver involution pro-metastatic ECM proteins, i.e., collagen and tenascin C, are deposited and the abundance of immature monocytes and macrophages is elevated [18]. Weaning-induced liver involution promoted breast cancer liver metastasis in a model where tumor cells were delivered to the liver via portal vein injection [18]. This observation of increased liver metastasis in postpartum mice, a so-called “involution advantage”, suggests that physiologic liver involution induces a metastatic niche. Of clinical relevance, liver metastases are common in younger breast cancer patients [19]. Further, one recent study demonstrated that young postpartum breast cancer patients are more likely to develop liver metastasis than age matched, as well as tumor stage and subtype matched, never-pregnant patients [18]. Here, we use weaning-induced liver involution as a model of a physiologic process that induces a metastatic niche, and investigate potential mechanisms by which the involution liver supports metastasis.

We report new evidence that reproductive stage of the liver differentially impacts tumor multiplicity and morphology, including mesenchymal and desmoplastic phenotypes. Further, we found that the normal process of liver involution establishes an adaptive immune suppressed environment, and that the involution metastatic advantage can be recapitulated with depletion of cytotoxic CD8 T cells. These data are evidence for reproductive stage dependent physiologic remodeling of the liver metastatic niche with implications for niche-targeted interventions.

## 2. Results

**Figure 1:**
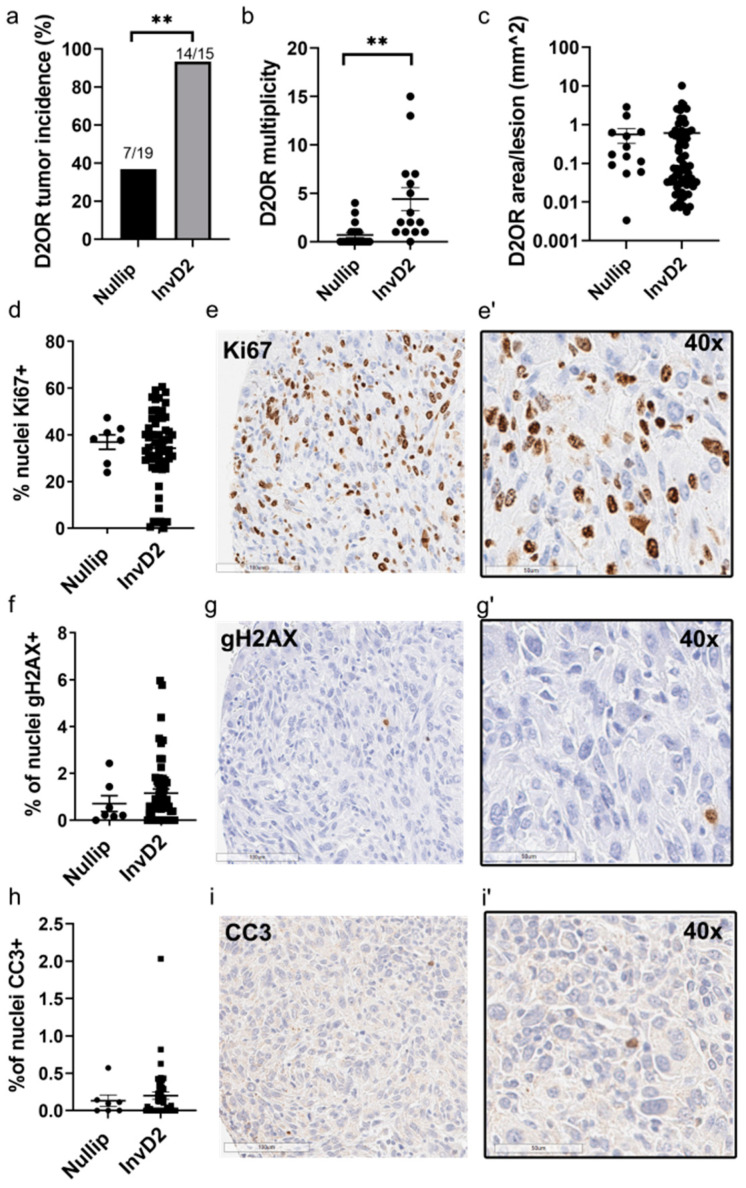
Increased metastasis in involution hosts does not associate with enhanced tumor growth. (**a**) Incidence of D2OR liver metastases in nulliparous (nullip, *n* = 19) and involution day 2 (InvD2, *n* = 15) mice, *n* = 3 independent studies; Two-sided Fisher’s exact test; (**b**) Number of metastases (i.e., multiplicity) per mouse in nullip (*n* = 19) and InvD2 (*n* = 15) groups; Two-tailed T test; (**c**) Area per tumor in nullip (*n* = 13 tumors) and InvD2 (*n* = 66 tumors) hosts; IHC quantification of percent of tumor nuclei positive for (**d**) Ki67 in nullip (*n* = 7 tumors) and InvD2 (*n* = 57 tumors); (**f**) γH2AX in nullip (*n* = 7 tumors) and InvD2 (*n* = 58 tumors), and (**h**) cleaved caspase 3 (CC3) in nullip (*n* = 7) and InvD2 (*n* = 48); Representative IHC images at low and high magnification of Ki67 (**e**,**e’**), γH2AX (**g**,**g’**), and CC3 (**i**,**i’**) stains. ** *p* < 0.01.

To model the clinical observation that young women with good prognostic breast cancers can progress to metastatic liver disease [20], we used a syngeneic BALB/c mouse model where mammary tumor cells of low metastatic ability, D2.OR cells [21,22], are delivered directly to the liver via the portal vein [23]. This allowed us to interrogate how liver involution may impact multiple steps of the metastatic process, including tumor cell extravasation into the liver, initial survival in the liver parenchyma, formation of micrometastases, and expansion into overt liver metastases. We first evaluated the formation of overt tumors at 6 weeks post-injection, and found a >2-fold increased incidence of liver metastases in mice injected at involution (InvD2) compared to mice injected at nulliparous (Figure 1a). Tumor multiplicity was also increased in the involution group (Figure 1b). We next asked if involution provides a growth advantage to these metastatic lesions. We found no differences between nulliparous and involution groups in tumor size (Figure 1c), tumor proliferation as measured by Ki67 (Figure 1d,e), nor death as measured by gH2AX or cleaved caspase 3 (Figure 1f–i). These data are consistent with the involution metastatic niche promoting tumor establishment rather than growth. Similar results of increased multiplicity in the absence of tumor size or proliferation advantage were observed in the involution group with D2A1 cells, a line with known high metastatic potential [21], suggesting similar mechanisms of promotion in two distinct cancer cell lines with different intrinsic metastatic capabilities (Figure S1). 

**Figure 2:**
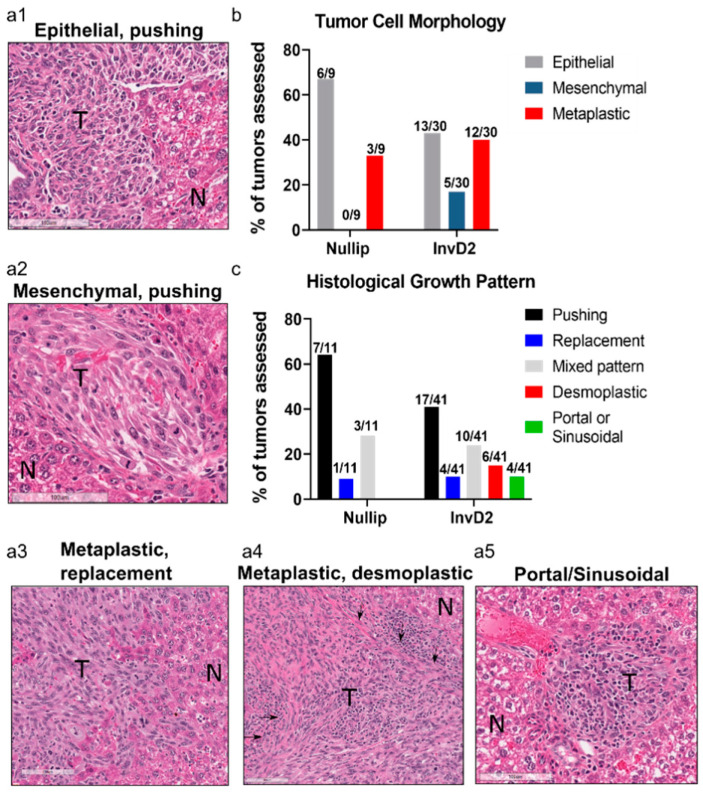
Histology of murine mammary liver metastases resembles human disease and shows increased histological heterogeneity in involution hosts. Representative hematoxylin and eosin stained images from D2.OR tumors classified as (**a1**) epithelial, pushing, (**a2**) mesenchymal, pushing, (**a3**) metaplastic, replacement, (**a4**) metaplastic, desmoplastic with desmoplastic areas noted by black arrows, and (**a5**) portal/sinusoidal pattern. Tumors are denoted by “T” and adjacent normal liver by “N”; (**b**) Quantitation of D2.OR tumor cell morphology and (**c**) histological growth pattern by host reproductive stage.

Clinically, metastases to the liver are categorized into distinct histologies, which have implications for disease outcome [24]. Utilizing histological criteria previously reported in humans, we evaluated tumor cell phenotypes as epithelial, mesenchymal, or metaplastic (i.e., heterogeneous tumors composed of both epithelial and mesenchymal regions with irregular nuclear morphology). We also characterized five previously defined human liver metastasis growth patterns: pushing, replacement, desmoplastic, portal/sinusoidal, and mixed patterns [24]. Representative images of the D2.OR mammary tumor cells growing within mouse livers are shown (Figure 2a1–a5). These murine liver metastases were heterogeneous and highly reminiscent of the tumor heterogeneity observed in human liver metastases. We found that reproductive state influenced both tumor cell morphology and growth pattern (Figure 2b,c). Specifically, tumors that evolved in the nulliparous host liver were more likely to be epithelial in morphology and have a pushing growth pattern. In contrast, tumors from the involution host liver showed greater diversity with increased mesenchymal cell morphology and heterogeneity in growth patterns including desmoplastic and portal/sinusoidal patterns. This observed conservation in liver metastases histology between mice and humans suggests increased human relevance of our breast cancer liver metastasis model. Further, these data represent the novel finding that tumor histology within the liver is shaped by reproductive state of the host, and are consistent with the hypothesis that the liver microenvironment can dictate tumor histology independent of intrinsic tumor cell biology. 

**Figure 3:**
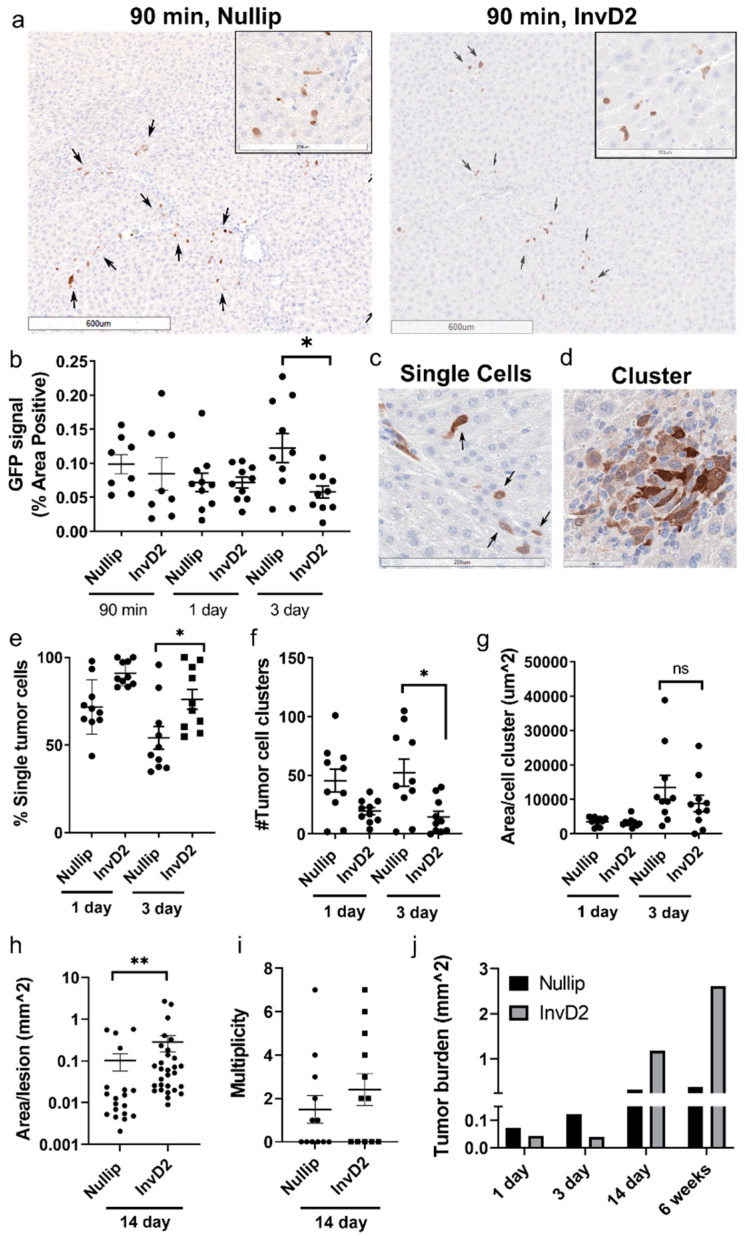
Involution metastatic advantage is observed by 2 weeks post-injection but not at earlier time points. (**a**) Representative low and high magnification IHC images of liver FFPE sections stained to identify GFP-tagged D2.OR cells, arrows indicate tumor cells; (**b**) IHC quantification of GFP+ tumor cells in liver FFPE sections from mice euthanized 90 min (*n* = 4 mice/group), 1 day (*n* = 5 mice/group), and 3 days (*n* = 5 mice/group) after intraportal injection of 500,000 D2.OR-GFP tumor cells per liver. Two sections >200µm apart were assessed per mouse to quantify independent tumor cells; One-way ANOVA; Representative high magnification images showing GFP+ tumor cells as (**c**) single cells (1 day time point) or, (**d**) clusters, defined as >3 tumor cells, i.e., micro-metastases, (3 day time point); Quantification of GFP+ (**e**) single tumor cells as a percent of total tumor cell signal, (**f**) number of tumor cell clusters, and (**g**) size of tumor cell clusters at 1 and 3 days post-injection; One-way ANOVA; IHC quantification of GFP+ (**h**) tumor area and (**i**) multiplicity from mice euthanized 14 days after intraportal injection of 500,000 D2.OR-GFP tumor cells; Two-tailed Mann-Whitney test; (**j**) Timeline of tumor burden in nullip (black) and InvD2 (grey) mouse groups, showing “switch” from early (3 day) advantage in nullip to later (≥14 days) advantage in involution. * *p* < 0.05, ** *p* < 0.01.

Because our data supports tumor cell establishment rather than growth as the metastatic advantage in the involuting liver, we predicted that there would be an increased abundance of tumor cells at early time points post-injection in involution hosts. Such data could be indicative of increased tumor cell extravasation and/or initial survival/proliferation advantage in the involuting liver environment. As a tool to visualized tumor cells before they grow into microscopically detectable lesions, we used GFP-tagged D2.OR tumor cells. Because GFP is a foreign protein, we utilized GFP-tolerant mice for these experiments, which were a gift from Lalage Wakefield (National Cancer Institute) [25]. Independent of reproductive state, by 90 min after tumor cell injection, we observed an even distribution of tumor cells throughout the liver, demonstrating uniform tumor cell dispersion (Figure 3a, arrows indicate tumor cells). We found no reproductive stage differences in tumor cell abundance at 90 min or one-day post-injection (Figure 3b), suggesting that the metastatic advantage in involution hosts is not due to differential tumor cell extravasation or initial survival. Unexpectedly, at three-days post-injection there was greater tumor cell abundance in the nulliparous host liver (Figure 3b), indicative that the liver microenvironments are indeed different between nulliparous and involution hosts. In sum, these data do not support the hypothesis that the metastatic advantage in the involution liver is due to increased tumor cell seeding and/or survival at early time points. 

We observed two distinct tumor cell patterns at these early time points: single tumor cells (Figure 3c) and tumor cell clusters (Figure 3d). When we delineated these patterns by reproductive group, there was an increase in single cells found in the involution host, as early as one-day post-injection (Figure 3e). An increase in tumor cell clusters in the nulliparous group was also evident as early as one-day post-injection (Figure 3f); however, there was no evidence of a growth advantage in either reproductive group, as measured by tumor area per cluster (Figure 3g). Since tumor cell clusters are consistent with a more epithelial-like state and single tumor cells with a more mesenchymal-like state [26], these early time point data may shed light on our six-week endpoint findings, where mesenchymal-dominant tumors were only observed in the involution group (Figure 2b). In sum, tumor cell morphology may be established early after tumor cell injection.

Although overt metastases are increased in the involution host liver at five to six weeks post-injection (Figure 1, Figure S1), we found no evidence for increased tumor signal in the involution group within three-days of tumor cell injection (Figure 3b). Therefore, we extended the study timeline to 14 days and found increased tumor cell signal as measured by area per lesion, multiplicity, and tumor burden in the livers of involution hosts (Figure 3h–j). Given that immune recognition and adaptive immune activation is known to take 7–10 days to develop after antigen exposure in viral systems [27], this 14 day timeframe of the involution metastatic advantage is consistent with an altered immune environment that impacts adaptive immune recognition of tumor cells. 

**Figure 4:**
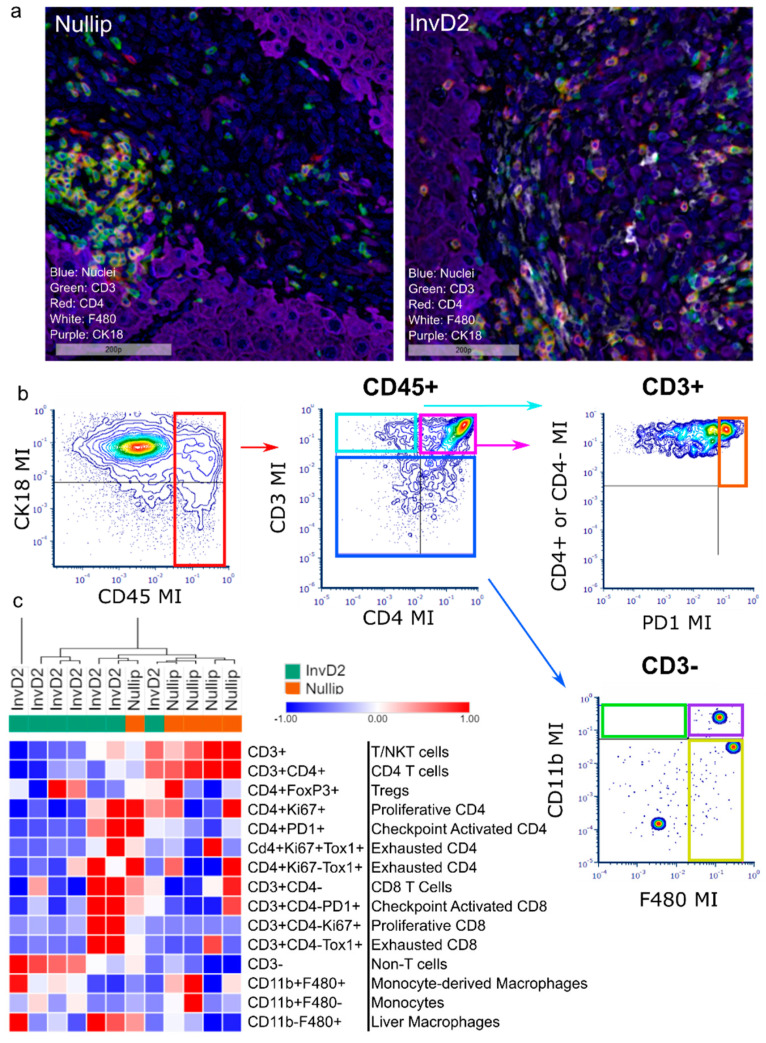
Immune milieu of liver metastases differs by host reproductive state. (**a**) Representative pseudo-colored multiplex IHC images of liver metastases in nullip and InvD2 mice euthanized 14 days after intraportal tumor cell injection showing select biomarkers: nuclei (blue), CD3 (green), CD4 (red), F480 (white), and CK18 (purple); (**b**) Representative image cytometry gating schema, showing the identification of CD45+, CD3+/−, CD4+/−, and PD1+ populations; (**c**) Hierarchical-clustered heat-map of intra-tumoral immune cell populations (%CD45+) identified by image cytometry of multiplex IHC staining for 9 biomarkers (CD45, CD3, CD4, FoxP3, Ki67, PD1, Tox1, CD11b, and F480). Tumor data are reported as average per mouse.

Given our prediction that immune cells are involved in the involution metastatic advantage, we sought to profile the intra-tumor immune milieu. We interrogated tumor-bearing livers from nulliparous and involution stage mice 14 days after intra-portal injection of D2.OR-GFP tumor cells, the earliest time point where the involution advantage was observed. CD45 staining revealed high levels of tumor infiltrating immune cells, but no differences between groups (Nullip: 12%, InvD2: 14%). Since immune cell abundance did not differ, we next asked whether the activation state of intra-tumoral CD4 or CD8 T cells differed by reproductive group, which could account for differential anti-tumor immune function. We stained for nine common leukocyte lineage and functional state markers (CD45, CD3, CD4, FoxP3, Ki67, PD1, Tox1, CD11b, and F480) using multiplex immunohistochemical (mIHC) methods. Representative pseudo-colored images of select biomarkers, including CK18 marking hepatocytes and some tumor cells, are shown (Figure 4a). Using image cytometry, we quantified various T cell and myeloid subsets as percentage of total CD45+ immune cells (Figure 4b) and performed hierarchical clustering by case (Figure 4c). We found that the profile of tumor immune infiltrate separated cases by reproductive stage of the host, with only one case each clustering outside of its reproductive group (Figure 4c). Of note, we found no relationship between tumor size and immune cell infiltrates (Figure S2), suggestive that tumor size alone does not dictate immune cell infiltrate in this model. In sum, these data support the idea that reproductive stage of the host shapes the composition of the intra-tumoral immune milieu with implications for anti-tumor immunity.

The mIHC analyses further revealed that overall, all of the metastatic tumors are “hot” as defined by high CD3+ cell infiltration (Figure 4a). The majority of CD3+ cells did not show signs of activation, such as the expression of Ki67 or PD1. There were two out of seven involution cases with CD3+ cells that expressed Ki67, PD1, and Tox1, potentially indicating immune activation with exhausted features [28,29]. We next investigated the intra-tumoral ratio of CD4+ to CD8+ T cells, since higher CD4:CD8 ratios associate with increased functional CD8+ T cell responses and better disease outcomes [30,31,32]. For these studies we define CD4 T cells as CD45+CD3+CD4+, and identify putative CD8 T cells as CD45+CD3+CD4−, herein referred to as CD8. We found higher CD4:CD8 ratios in tumors from nulliparous mice compared to tumors in involution mice (37:1 vs. 8:1). Finally, while CD3+ and CD3+CD4+ T cells were enriched in tumors from nulliparous hosts, CD45+CD3− (putative myeloid lineage cells) and F480+ cells (mature macrophages) were greater in tumors from involution hosts. Taken together, these data raise the possibility that the immune milieu of involution group tumors is characterized by low CD4 and high myeloid populations, which could result in impaired anti-tumor cytotoxic immunity. 

**Figure 5:**
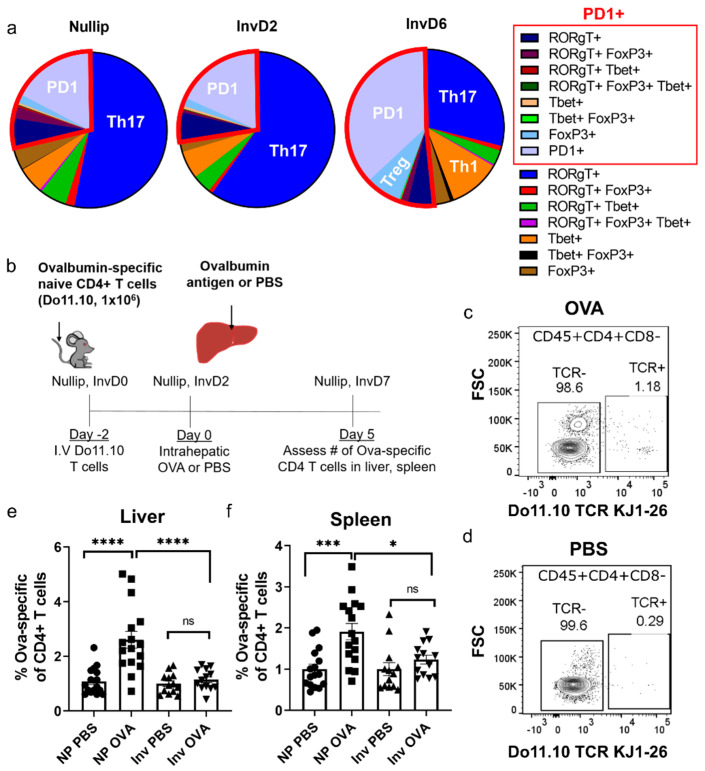
CD4+ T cell polarization is reproductive stage dependent with implications for antigen-specific activation. (**a**) Flow cytometry phenotyping of CD45+ CD4+ T cells from livers of mice at nullip, InvD2, and InvD6 reproductive stages (nullip *n* = 8, InvD2 *n* = 5, InvD6 *n* = 6). Only CD45+ CD4+ T cells that expressed at least one of the four phenotyping markers RORgT, Tbet, PD1, or FoxP3 are shown (see Figure S3 for total CD45+CD4+ T cell polarization); (**b**) Experimental schema for the in vivo T cell activation assay (Figure S4 shows transferred CD4+ Do11.10 cells); Representative flow cytometry plots showing expression of ova-specific Do11.10 T cell receptor (TCR) in CD45+ CD4+ CD8- cells from mice intra-hepatically injected with (**c**) ovalbumin (OVA) antigen or (**d**) PBS; Flow cytometry quantification of ova-specific CD4+ T cells (Do11.10 TCR+) as %CD4+ in (**e**) whole liver or (**f**) spleen across 2 independent experiments (nullip PBS *n* = 16, nullip OVA *n*=15, InvD2 PBS *n* = 13, InvD2 OVA *n* = 13). Data normalized to PBS average for reproductive group, One-way ANOVA. * *p* < 0.05, *** *p* < 0.001, **** *p* < 0.0001.

A defining attribute of tumors in the involution group is that they evolved under unique micro-environmental conditions. Liver involution involves programmed cell death of hepatocytes and wound-healing like processes [18], which in other contexts are known to elicit suppression of cytotoxic adaptive immune response [33,34]. This is because phagocytosis of dying cells induces tolerogenic antigen presenting cell functions, which limit autoimmune reactions to the dying cell [33,34]. Based on these prior studies, we hypothesized that the liver metastatic niche during involution may be characterized by immune tolerance, which inadvertently permits tumor cell evasion. To address this hypothesis, we phenotypically assessed CD4+ T cells by flow cytometry at nulliparous, involution day 2, and involution day 6 with the prediction that T cell polarization would be dependent on reproductive state. We quantitated CD4+ T cells for expression of Th1, Th17, or regulatory (Treg) transcription factors, as well as PD1 (Figure 5a, Figure S3). Each reproductive stage showed a large population of RORgT+ T cells, which we classify as Th17-skewed; however, by involution day 6 (InvD6) this Th17 population had reduced prominence. At InvD6 we also found increased Tbet, PD1, and FoxP3+ CD4 T cells (Figure 5a), data consistent with inflammation (Tbet, Th1), T cell activation (PD1) and induction of a regulatory state (FoxP3+, Treg) in the normal, involuting liver. 

One feature of immune tolerance is the inability of adaptive immune cells to proliferate in response to their cognate antigen, which can be robustly assessed in functional assays. To this end, we used an in vivo CD4+ T cell activation assay where we adoptively transferred ovalbumin antigen-specific CD4+ T cells (Do11.10, Figure S4) systemically and subsequently injected their cognate antigen (OVA) into the liver via intrahepatic injection (Figure 5b). Five days after ova or phosphate buffer saline (PBS) control injection, ova-specific T cells were assessed by flow cytometry in both the liver and spleen as a measure of T cell activation. Representative flow cytometry plots are shown (Figure 5c,d, Figure S5). Firstly, we found that the nulliparous host liver responds to ova antigen with a 2-fold increase in ova-specific T cells compared to PBS controls (Figure 5e). Conversely, in the involution group there was no increase in ova-specific T cells after exposure to ova antigen compared to PBS, providing functional evidence for immune tolerance. Similar results were observed in the spleen (Figure 5f). These data raise the possibility that deficient adaptive immunity contributes to increased liver metastasis in the involution host, while intact adaptive immunity is partially responsible for limiting liver metastasis in nulliparous hosts.

**Figure 6:**
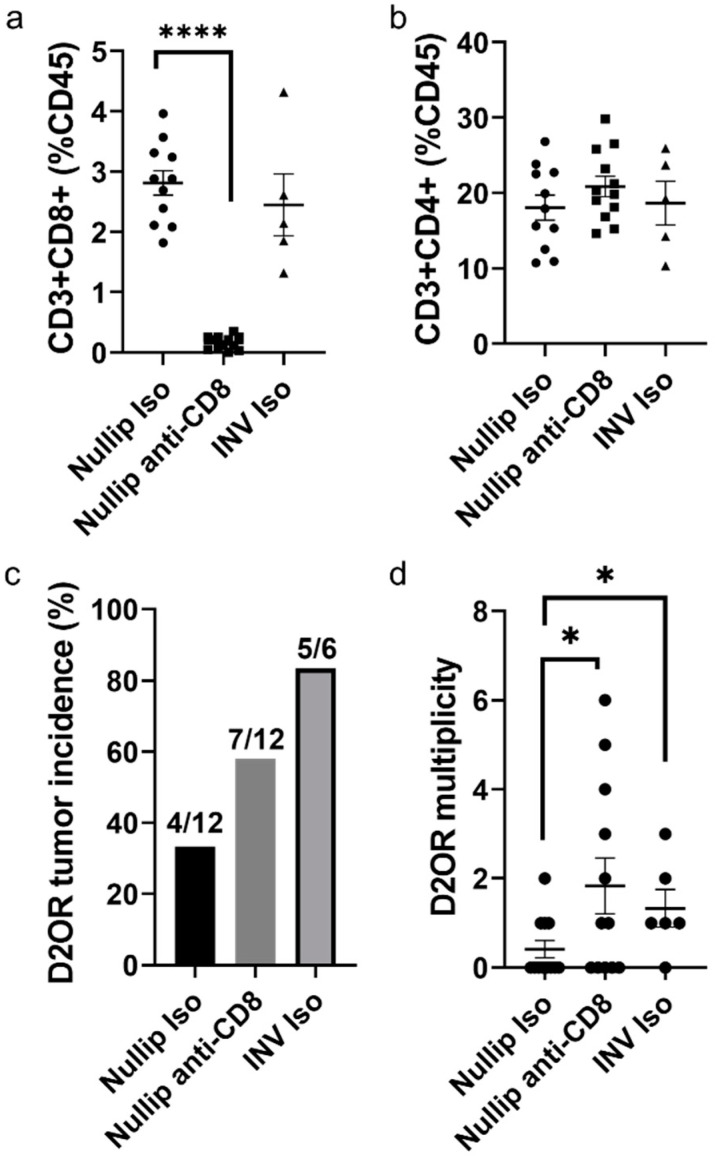
Depletion of CD8+ T cells in the nulliparous host recapitulates the involution metastatic advantage. Flow cytometry quantification of (**a**) CD8 and (**b**) CD4 T cells from livers of mice at endpoint of 6 week metastases study where mice were treated with CD8-depleting or isotype control antibody demonstrating effective CD3+CD8+ T cell depletion; (**c**) Incidence and (**d**) multiplicity of D2.OR liver metastases 6 weeks after intraportal injection of 50,000 D2.OR tumor cells with and without CD8 depletion in nulliparous hosts. Nullip iso *n* = 12, Nullip anti-CD8 *n* = 12, INV iso *n* = 6. One-way ANOVA. * *p* < 0.05, **** *p* < 0.0001.

If an adaptive immune response to tumor cells in nulliparous hosts contributes to reduced liver metastasis, then we expect depletion of CD8+ T cells to increase metastasis and possibly recapitulate the metastatic advantage observed in the involution group. In our portal vein liver metastasis model, nulliparous mice were either CD8+ T cell-depleted via intraperitoneal injection of anti-CD8 antibody (BioXcell clone 2.43, Lebanon, NH, USA) or treated with isotype control. Antibody dosing began two days prior to tumor cell injection (0.2 mg initial dose) and continued every four days for the course of the experiment (0.1 mg maintenance dose). This antibody dosing scheme was designed to minimize antibody concentration and effectively deplete CD8+ T cells in the liver, in order to mitigate possible off-target antibody effects (Figure S6). Depletion of CD8+ T cells (CD45+CD3+CD8+CD4−) in the liver was confirmed at study endpoint by flow cytometry with no discernable effect on CD4+ T cells (CD45+CD3+CD4+CD8−) (Figure 6a,b). Incidence and multiplicity of liver metastasis six weeks after injection increased in anti-CD8 treated nulliparous animals compared to isotype control (Figure 6c,d). Of note, multiplicity in the anti-CD8 treated nulliparous group was commensurate with multiplicity in the isotype treated involution group. These data are consistent with differential CD8+ T cell activation during involution contributing to the increase in metastatic outgrowth and multiplicity observed in the involution group.

## 3. Discussion

Metastasis has historically been considered an intrinsic property of the cancer cell [35,36,37,38], which is in part enabled by a cancer cell’s ability to reshape distant sites into favorable environments [6,7,10,14]. In line with this thinking, tumor-induced remodeling of the liver microenvironment has been well described [39,40]. Such tumor-education of the liver pre-metastatic niche includes macrophage and fibroblast activation that results in pro-metastatic inflammatory signaling and ECM protein deposition [11,12,13]. However, recently it has been posited that pro-metastatic liver niche remodeling could occur independent of a primary tumor, including processes such as liver repair and regeneration following injury [18,41]. Here, we provide further evidence of a physiologic process, weaning-induced liver involution, which induces a liver pre-metastatic niche. 

Previously, it was demonstrated that liver metastases were promoted in postpartum mice using the fast-growing, metastatic-competent D2A1 mouse mammary tumor cell line [18]. In the present study, we find that the postpartum liver also supports metastasis of D2.OR mammary tumor cells, a cell line with lower growth rate and demonstrated low metastatic potential [21,22]. An advantage of using a cell line with slow growth is that it may permit evolution of tumor cell-microenvironment interactions that are more reminiscent of what occurs in human disease. 

Liver metastases can be categorized by growth pattern in relation to adjacent normal hepatocytes using a method developed for colorectal cancer liver metastases [24]. Three main growth patterns have been identified: desmoplastic, pushing, and replacement. Patients whose metastases had a dominant desmoplastic pattern were found to have longer overall and recurrence free survival compared to those with a dominant replacement growth pattern [24,42]. In our study, we classified mouse mammary cancer liver metastases according to these guidelines. We found potential relevance to human disease, as the heterogeneous histological growth patterns reported in human liver metastases were observed. Use of this histological growth classification for liver metastases is not common in breast cancer. However, a recent study of 58 breast cancer patients found that the replacement pattern was dominant, and as in colorectal cancer liver metastasis, the desmoplastic pattern associated with longer overall survival [43]. Although we found approximately 14% of tumors with desmoplastic growth pattern in murine involution hosts and none in nulliparous hosts, our study was not designed to evaluate survival so we cannot determine survival differences between tumor histologic patterns. While a key strength of our breast cancer liver metastasis model is the observation of a range of tumor histological patterns in the liver, how reproductive state influences these patterns and outcomes will require further investigation. 

Tumor cell morphological properties such as cytoplasmic volume, roundness, and elongation have been implicated in metastatic potential [44,45]. These studies viewed tumor cell morphology as a cell-autonomous property, yet the microenvironment may also shape morphology [46,47]. Here, we observed distinct tumor cell morphological patterns based on host reproductive stage. Single, solitary tumor cells were more prevalent in the involution group. The high percent of single tumor cells in the involution host could be indicative of a more mesenchymal phenotype since they lack cell-cell adhesion with other tumor cells and show elongated morphology. In contrast, tumor cell clusters, indicative of tumor epithelial cell interactions, were increased in nulliparous host livers. Further, we have reason to believe the involution liver could preferentially support tumor cell epithelial-to-mesenchymal transition (EMT). The involution liver has significantly elevated TGFβ and ECM proteins tenascin-C and fibronectin, which are all known to mediate EMT [18,48,49,50]. Promotion of tumor cell EMT by the involution microenvironment is intriguing, given the connection between mesenchymal state and metastatic promotion [50], including evasion of the immune system [51,52,53,54]. Our work does not directly test the potential of tumor clusters versus single cells in the liver environment to form overt metastases. However, our data are consistent with an advantage to the solitary tumor cells since there are more metastatic events in the involution group at study endpoint. Additional studies are needed to explore the relationship between the presence of early single tumor cells and metastatic success in this model. 

We show in both D2.OR and D2A1 models that liver metastatic advantage involves increased metastasis incidence and multiplicity in involution hosts, which appears to occur without a tumor cell proliferation advantage. Seeing no proliferation advantage, we predicted that the involution microenvironment increases a tumor cell’s chance of successfully forming a metastasis, and used the metastatic cascade as framework to investigate this possibility [9,10]. The metastatic cascade describes how a tumor cell escapes the primary site and arrives at a secondary organ, then must lodge, extravasate, survive, form micrometastasis, and grow in order to become a metastatic tumor [9,10]. Prior work has shown that late stages (i.e., after arrival at the secondary organ) are rate-limiting for metastatic success [55,56]. 

We initially hypothesized that the involution advantage would be evident early, at the tumor cell extravasation, survival, and/or proliferation steps, due to the known, pro-metastatic ECM remodeling that occurs in the liver post wean [18]. The rodent involuting liver is enriched for collagen I, fibronectin, and tenascin-C [18,57], ECM proteins demonstrated to promote establishment of tumor cells in the niche [58,59,60,61,62]. However, we found no evidence for involution-specific ECM proteins contributing to these early, metastatic events. Specifically, no differences in tumor cell abundance were observed between groups at days one and three post portal vein injection, time points when tumor cells become established in the niche. Rather, the involution metastatic advantage was observed at 14 days after tumor cell injection. This timeframe is consistent with the involuting liver promoting a later step in the metastatic cascade, at the transition from a micro- to an overt metastatic lesion. 

Suppression of anti-tumor immunity is one potential mechanism by which the involuting liver promotes tumor growth from micro- to overt metastasis. Others have shown that the liver pre-metastatic niche is characterized by alteration to innate immune composition, including elevated neutrophils, bone marrow-derived myeloid cells, and M2-polarized macrophages [40,63]. Adaptive immune cells, including CD4+ Th17 polarized and T regulatory (Treg) cells, have also been shown to play a functional role in promoting liver metastasis [64,65]. These innate and adaptive immune cell populations support metastasis in part by limiting anti-tumor immunity via active suppression of the cytotoxic T cell response [66]. In previous work from our lab, we showed that the involution liver has increased abundance of neutrophils, immature monocytes, and macrophages compared to the nulliparous liver [18]. Here we build on that dataset to show that CD4+ Th17 polarized T cells are abundant in the murine liver regardless of reproductive state. Furthermore, CD4+ T cell polarization is modulated by reproductive state with the normal, involuting liver being characterized by Th1-skewed inflammation, upregulation of the checkpoint molecule PD1, and increased abundance of Tregs. This immune composition data demonstrate that the involution liver has most of the immune characteristics attributed to a tumor-educated pre-metastatic niche [63]. Further, utilizing a functional in vivo T cell activation assay, we identify immune tolerance as a new attribute of weaning-induced liver involution, similar to what was recently described in the mammary gland during weaning-induced involution [67]. 

We propose that the stimulus for tolerance mechanisms is the hepatocyte programmed cell death that occurs post-wean to return the pregnancy and lactation-enlarged liver to its pre-pregnant size [18]. Such programmed cell death is considered “immunologically silent” [33,34]. This immune-tolerization is achieved in part via signaling from the apoptotic cell to the antigen presenting cell (APC), which results in reduction of the co-stimulatory signals on the APC typically required for T cell activation [67,68]. An additional, unexplored possibility is that the unique ECM composition of the involuting liver contributes to regulation of the immune milieu. It is known that collagen, fibronectin, and tenascin-C can regulate immune cell trafficking and function [69], including recruitment of immature monocytes [13] and suppression of T cell activation [70]. These published reports provide further rationale for the matrisome of the involuting liver contributing to impaired anti-tumor immunity, although such a possibility requires future study. In sum, these studies of normal involuting liver provide evidence for both T cell inflammation and tolerance, with implications for tumors emerging in this environment.

Clinically, tumors that are classified as “hot” (i.e., those with robust T cell infiltration) are associated with better prognosis, most likely due to anti-tumor effects of CD3+ T cells [71,72]. In our murine model of breast cancer liver metastasis, we find that the majority of tumors were CD3+ “hot”, indicating that T cells were able to enter tumors in both nulliparous and involution hosts. While robust intra-tumoral CD3+ infiltration is typically a positive biomarker for disease prognosis, in our mouse model the majority of tumors in involution hosts and ~1/3 of tumors in nulliparous hosts grew into large metastatic lesions. This paradox could indicate that the effector status of CD3+ cells is differentially compromised by reproductive state, a premise supported by the observation that nulliparous and involution tumors delineated by immune profiling. Specifically, tumors that evolved in involution host livers had lower CD4:CD8 ratios, increased myeloid infiltration, and increased exhaustion markers including PD1 and Tox1 [28,29]. Our finding that depletion of CD8+ T cells in the nulliparous host recapitulates the involution metastatic advantage provides further rationale for the hypothesis that ineffective anti-tumor immunity contributes to the involution metastatic advantage. Whether liver metastases in young women’s breast cancer show similar pro-tumor patterns of tumor immune infiltrates remains to be determined. 

The results presented here may have significance for a recently appreciated type of aggressive, young women’s breast cancer called postpartum breast cancer (PPBC) [73,74,75]. A diagnosis of breast cancer within five years of a recent pregnancy is an independent predictor of liver metastasis, which suggests that the postpartum liver may be uniquely susceptible to metastasis as we demonstrate in the rodent models [18]. In support of this hypothesis, we recently found that the size of the human liver is regulated by reproductive state and provided the first data suggesting that weaning-induced liver involution may occur in women [76]. Since we report in the rodent model a relationship between immune composition in the normal involution liver and resulting tumor immune infiltrate, such a paradigm may also exist in human breast cancer liver metastasis. If so, the rodent studies provide evidence that PPBC liver metastasis may involve immune modulation and be responsive to immune checkpoint therapies. While checkpoint blockade immunotherapy must be tested in preclinical models, our findings could help direct much-needed new treatments for breast cancer liver metastases in young women.

## 4. Materials and Methods 

### 4.1. Animal Husbandry

Oregon Health & Science University (OHSU) Institutional Animal Care and Use Committees approved (TR01_IP00000967, approved on 13 April 2020) all animal procedures. Age-matched (10–12 weeks) female BALB/c mice (Charles River Laboratories, Wilmington, MA, USA) were housed and bred as described [77]. Briefly, for involution group animals pup number was normalized across dams to assure equal lactation and pups were weaned between 9-11 days after birth. At study endpoints, mice were euthanized across groups either by CO2 asphyxiation or while under anesthesia by exsanguination via portal vein perfusion with PBS. Whole livers and/or spleens were removed, washed 3× in 1× PBS, and processed for subsequent assays as described. 

### 4.2. Cell Culture 

D2.OR, D2.OR-GFP, and D2A1 mouse mammary tumor cells were kindly provided by Ann Chambers (University of Western Ontario, London, Ontario, Canada) and cultured as previously described [21]. For tumor cell injection preparation, cells were washed and suspended in cold 1× PBS. Cells were tested in January 2018, were confirmed murine pathogen and Mycoplasma free, and the origin of cells was validated (Idexx Bioresearch, Columbia, MO, USA). Cells used in these studies were within 3 passages of tested vials. 

### 4.3. Liver Metastases Studies

Liver metastases were induced by intra-portal vein injection of D2OR, D2OR-GFP, and D2A1 mouse mammary tumor cells, as previously described [23]. Tumor cells were suspended in 10 µL 1×PBS with various number of cells: 50,000 D2.OR for 6 week endpoint and CD8 T cell depletion studies; 5,000 D2A1 for 5 week endpoint study; 500,000 D2.OR-GFP for 90 min, 1, 3, and 14 day endpoint studies. Tumor cells were injected into either involution group mice 2 days after weaning, involution day 2 (InvD2), or age-matched nulliparous mice. For D2A1 and D2.OR studies, BALB/c mice were used. For D2.OR-GFP studies, “Glowing Head” BALB/c mice (Gnrhr-luc/EGFP) were used as these mice are tolerant to the GFP protein, which were a gift from Lalage Wakefield (National Cancer Institute, USA) [25]. Whole liver was formalin-fixed and paraffin embedded (FFPE) for histological analyses. Liver metastases were assessed following euthanasia at 5 and 6 week time points by visual assessment of the liver and by hematoxylin and eosin staining of FFPE liver sections. For the D2.OR-GFP 90 min, 1, 3, and 14 day endpoint studies, immunohistochemistry (IHC) for GFP+ tumor cells was used for assessment. To assess for solitary tumor cells and micrometastases, two distinct depths of liver tissue ≥200 µm apart were sectioned per mouse for subsequent IHC analyses. 

### 4.4. CD8 Depletion Experiment

Antibody concentration (BioXcell; clone 2.43) for CD8+ T cell depletion in the liver and spleen was determined by testing two initial dose concentrations (0.2 mg, 0.4 mg) and two maintenance dose concentrations (0.1 mg, 0.2 mg). Antibody was delivered in 200 µL sterile PBS via intraperitoneal injection, with initial dose given at day 0 and maintenance doses given at day 4 and day 8. 72 h post last maintenance dose mice were euthanized and liver and spleen collected for flow cytometry assessment. For subsequent tumor experiments the lowest concentrations combination that was effective at depleting CD8+ T cells was used: 0.2 mg initial dose and 0.1 mg maintenance dose (Figure S4). Mice were randomized, in a rolling study design, to receive either isotype control (BioXcell; BE0090) or CD8 depleting antibody (BioXcell; clone 2.43). In tumor studies, initial antibody dose was given 2 days prior to tumor cell injection and maintenance doses were given every 4 days for the 6 week course of the experiment. Data are presented as percent of CD45+ cells. 

### 4.5. Histological Analyses

Liver FFPE tissue sections representing all liver lobes were stained with hematoxylin and eosin to evaluate for presence of tumors, tumor size, tumor multiplicity, and tumor morphology. Morphology was characterized as epithelial-dominant, mesenchymal-dominant, or metaplastic, defined as tumors with irregular nuclei and a mix of epithelial and mesenchymal components. Histological growth pattern was characterized as established for scoring liver metastases in colorectal cancer into pushing, replacement, desmoplastic, mixed pattern, and portal/sinusoidal patterns [24]. Assessments were completed by two evaluators blinded to study design.

### 4.6. Immunohistochemistry

Single-stain IHC detection was performed as described [78]. Briefly, tissues were deparaffinized, rehydrated, and heat-mediated antigen retrieval was performed with EDTA for 5 min at 125 °C. The following primary antibodies were applied for 1 h at room temperature: Ki67 (1:400, Neo-markers #RM-9106-S), phospho-γH2AX (1:400, Cell Signaling #9718), Cleaved caspase 3 (CC3, 1:150, Cell Signaling #9664). Green fluorescent protein was applied overnight at 4 °C (GFP, 1:400 Abcam #ab13970). Secondary antibodies were applied for 30 min at room temperature: anti-rabbit (RTU, Agilent #K400) for Ki67, phospho-γH2AX, and CC3. For GFP, anti-chicken secondary was applied for 1 h at room temperature (1:1000, LSBio, LS-C61278). DAB chromogen (Agilent, K346889-2) with hematoxylin counter stain (Agilent, S330130-2) was used to visualize positive stain. Stained sections were scanned using the Aperio AT2 slide scanner (Leica Biosystems, Wetzlar, Germany). Signal quantification was performed by Aperio ImageScope v12.1.0.5029 as described previously [79]. For quantitation, all mice and tumors found in the stained tissue section were included for every analysis, unless staining was unsuccessful as defined as loss of tissue from slide and/or failure for positive controls to stain. All analyses were done by investigators blinded to study group. Data are presented as percent area positive unless otherwise noted in the figure legend.

Multiplex IHC staining was performed as previously described with modification [80]. Tissues were deparaffinized, rehydrated, and heat-mediated antigen retrieval was performed with EDTA for 5 min at 125 °C. Hematoxylin staining was performed on all tissues prior to antibody cycles, and tissues were scanned using the Aperio AT2 slide scanner (Leica Biosystems). Table 1 lists the antibodies and conditions used. Secondary antibodies were applied for 30 min: anti-rabbit (Histofine 414341F) and anti-rat (Histofine, 414311F). After each antibody cycle, tissues were scanned using the Aperio AT2 slide scanner (Leica Biosystems). Following scanning, 3-Amino-9-Ethylcarbazole (AEC) chromogen was removed with 1 × 70% and 1 × 100% alcohol wash for 2 min each. Primary and secondary antibodies were removed using 20% SDS-glycine pH 2 at 70 °C for 30–60 min. Secondary only and isotype controls were utilized to confirm antibody stripping after each cycle. This cycle was repeated for each primary antibody listed in Table 1. Each tissue slide included a positive control tissue microarray (TMA) to confirm primary antibody staining for each cycle. The TMA was comprised of mouse spleen, lymph node, and liver tissue from mice subjected to inflammatory stimuli. 

### 4.7. Multiplex IHC, Image Processing and Data Analysis

After staining, scanned images from each cycle were analyzed using an image processing pipeline previously described with minor modification [80]. Image alignment and extraction were performed using the SURF algorithm in the Computer Vision Toolbox of Matlab version R2018b (The MathWorks, Inc, Natick, MA, USA). Single cell segmentation and color deconvolution were performed in FIJI, and mean intensity quantification was performed in Cell Profiler version 3.5.1. Image cytometry was performed using FCS Express 6 Image Cytometry RUO (De Novo Software, Glendale, CA, USA). Data are presented as a percentage of CD45+ cells per tumor, averaged by mouse.

### 4.8. Flow Cytometry 

For flow cytometric quantification of liver CD4+ T cell polarization, left and caudate liver lobes were dissected following CO2 euthanasia and rinsed in 1× PBS to wash exterior blood. Liver lobes were minced and digested in 1 mg/mL collagenase I, 0.5 mg/mL hyaluronidase, and 0.5 mg/mL DNase at 37 °C for 30 min while rotating, and filtered through a 100 μm filter. Red blood cells were lysed using 1× RBC lysis buffer (eBioscience). Cell pellets were washed with 1× PBS, resuspended, and cells counted in trypan blue using a hemocytometer. 1 × 10^6^ cells per sample in 100 µL PBS or FACS buffer were blocked with CD16/32 (eBioscience, 1:100) for 30 min, stained for cell surface markers (Live/Dead, Aqua; CD45, 30-F11, PerCP; CD11b M1/70 BV711; CD4 RM4-5, BV786; PD-1 29F.1A12 PE-Cy7) for 30 min at room temperature, stained for intracellular proteins (Tbet 4B10, BV421; RORγT AFKJS-9, PE; FoxP3 FJK-16s, APC) overnight at 4C, and fixed with 4% paraformaldehyde (BD Biosciences, San Jose, CA, USA) for 20 min. Samples were ran on the LSRFortessa (BD Biosciences; Oregon Health and Science University) and analyzed as described below. Data in Figure 5 are presented as % of CD4+ cells that expressed at least one of the following: Tbet, RORγT, FoxP3, or PD1. Data in supplemental Figure S3 are presented as percent of total CD4+ cells.

For adoptive transfer experiment, whole liver and spleen were used for flow cytometric quantification of Do11.10 CD4+ T cells. Here a different digestion protocol with the addition of a Percoll gradient was used in order to enrich for immune cells. Whole livers were cut into small pieces and digested with 0.5 mg/mL collagenase 1 and 0.5 mg/mL DNase in RPMI1640 at 37 °C for 30 min, rotating. Tissue digests were filtered through a 100 μm filter and washed in RPMI1640. Samples were fractionated in a 33% Percoll solution with centrifugation at 800× *g* for 30 min at room temperature with no brake. Supernatant was collected and red blood cells were lysed using 1× RBC lysis buffer (eBioscience). Samples were washed and resuspended in RPMI1640 and counted in trypan blue using a hemocytometer. The entire liver sample was advanced to flow cytometry staining. For spleen digestion, spleens were processed through a 70 μm filter. Red blood cells were lysed using 1× RBC lysis (eBiosciences). Samples were washed and resuspended in PBS, counted in trypan blue, and 1 × 10^6^ cells per sample in 100 µL were stained for flow cytometry. The following antibodies were used: Live Dead (Aqua), CD4 (RM4-5, BV786), CD45 (30-F11, PerCP), Do11.10 TCR (KJ1-26, PE-Cy7), CD8 (53-6.7, APCe780). Samples were ran on the LSRFortessa (BD Biosciences; Oregon Health & Science University) and analyzed as described below. Data are presented as % of CD4+ cells.

For the CD8 depletion experiment, the median lobe was digested as in the adoptive transfer experiment and used to confirm CD8 depletion. However, if there was a tumor visually apparent in the median lobe at time of euthanasia, the caudate lobe was used to confirm CD8 depletion. The following antibodies were used: CD49b (DX5, BV421), CD3 (17A2, BV785), CD4 (RM4-5, FITC), CD8b (H35-17.2, PE-Cy7), CD45 (30-F11, APC), and Live/Dead (NearIR). Due to restrictions imposed by Covid-19, samples for the CD8 depletion study were ran on the Fortessa X50 (BD Biosciences; Fred Hutch, Seattle, WA, USA). One sample from the INV iso group had insufficient cellularity and was excluded from analysis. Data are presented as % of CD45+ cells.

All samples were analyzed using FlowJo v10 software (Becton, Dickinson & Company Franklin Lakes, NJ, USA). Unstained, single color, and fluorescent-minus-one staining controls were utilized for every experiment.

### 4.9. Adoptive Transfer In Vivo T Cell Activation Assay

T cell activation was assessed with an in vivo activation assay as described with minor modification [63]. Briefly, CD4+ splenocytes were isolated from Do11.10 transgenic female mice (Jackson Laboratories Stock #003303) and enriched to >95% CD4+ T cells (Figure S4) using a CD4+ negative selection kit (MACS miltenyi) under non-stimulating conditions (putative naïve T cells). Subsequently, 1 × 10^6^ isolated T cells in 100 µL 1× PBS were adoptively transferred via tail vein with insulin syringe into syngeneic BALB/c involution Day 0 or nulliparous age-matched hosts. Two days later either ovalbumin protein antigen (10 µg in 10 µL, Worthington) or 10 µL 1× PBS was injected into the left lobe of the liver via intrahepatic injection. Five days post antigen or PBS injection, whole liver and spleens were harvested, digested and stained for flow cytometry to detect Do11.10 T cells, as described above. A known quantity of absolute counting beads were added to samples (C36950 Invitrogen) and flow cytometry performed. Do11.10 TCR+ CD4+ T cell counts in liver and spleens were calculated by normalizing to the known abundance of counting beads. Data are presented as % of CD4+ cells.

### 4.10. Statistical Analysis and Hierarchical Clustering

Statistical analyses were performed using GraphPad Prism v.8 or v.9 software (GraphPad Software, San Diego, CA, USA). All data are expressed as mean ± standard error of the mean (SEM) unless otherwise noted. Statistical significance was determined using the following statistical tests with specific test fully described in the figure legends: Two-sided Fisher’s exact test, Two-tailed *T* test, One-way ANOVA with multiple comparisons, and Two-tailed Mann-Whitney test. Hierarchical clustering of multiplex IHC data was performed with the Morpheus web-based tool (https://software.broadinstitute.org/morpheus, accessed date: 27 January 2021), as described previously [81].

## 5. Conclusions

Here we provide the first evidence for reproductive state dependent CD4+ T cell activation in the normal murine liver, including induction of tolerance during weaning-induced liver involution. Further, we show that the immune milieu of mammary tumors evolving within the involuting liver microenvironment are durably altered in a manner consistent with tumor promotion. Our data supports the hypothesis that ineffective anti-tumor immunity within the involuting liver contributes to increased breast cancer liver metastasis. Taken together, our data provide a compelling argument for host reproductive factors being determinative for liver metastasis in the postpartum period with specific implications for young women’s breast cancer. Further, weaning-induced liver involution may serve as a robust model to investigate initiation and treatment of liver metastasis with potential utility for liver metastases overall.

## Figures and Tables

**Table 1:** Multiplex IHC Antibodies and Conditions.

Primary Antibody	Manufacturer	Catalog #	Lot#	Concentration	Incubation	Secondary Antibody
CD4	Cell Signaling	ab25229	Lot:4	1:50	O/N 4 °C	anti-Rb
CD45	BDPharminigen	550539	Lot: 4141820 & 9301732	1:50	60 min	anti-Rt
Ki67	Cell Signaling	12202	Lot: 6 (11/20)	1:800	60 min	anti-Rb
FoxP3	eBioscience	14-5773-82	Lot:E023634 + 2172602	1:100	60 min	anti-Rt
CD3	Abcam	ab16669	Lot: GR291605-1	1:100	60 min	Anti-Rb
Tox1	Abcam	ab237009	Lot: GR3241900-3	1:300	60 min	anti-Rt
PD1	Cell Signaling	84651	Lot: 4 (11/20)	1:200	60 min	anti-Rb
CD11b	Abcam	ab133357	EPR1344	1:30k	60 min	anti-Rb
F480	Cell Signaling	70076S	Lot:	1:500	60 min	anti-Rb
CK18	Abcam	ab181597	Lot: GR321105-11	1:1000	60 min	anti-Rb

## Data Availability

The data presented in this study are available in this article and in the supplemental material.

## Supplementary Materials

The following are available online at https://www.mdpi.com/article/10.3390/cancers13071698/s1, Figure S1: Increased D2A1 metastasis in involution hosts does not associate with enhanced tumor growth, Figure S2: Tumor immune infiltrate does not correlate with tumor size, Figure S3: CD4+ T cell polarization is reproductive stage dependent, Figure S4: Representative flow cytometry plots demonstrate enrichment of CD4+ cells from whole spleen for adoptive transfer in vivo T cell activation experiment, Figure S5: Representative flow cytometry gating for adoptive transfer in vivo T cell activation experiment, Figure S6: Evidence that lowest initial and maintenance dose concentrations of CD8-depleting antibody effectively depletes CD8+ T cells in the liver and spleen.
