# Supplementary material for: Immune Milieu Established by Postpartum Liver Involution Promotes Breast Cancer Liver Metastasis
Source: Cancers (Basel). 2021 Apr 3;13(7):1698. doi: 10.3390/cancers13071698 (PMC8038410; doi:10.3390/cancers13071698)
Supplement: Supplementary file 1 [file cancers-13-01698-s001.pdf]

Supplementary Materials: Immune Milieu Established by Postpartum Liver Involution Promotes Breast Cancer Liver Metastasis

Alexandra Q. Bartlett, Nathan D. Pennock, Alex Klug and Pepper Schedin

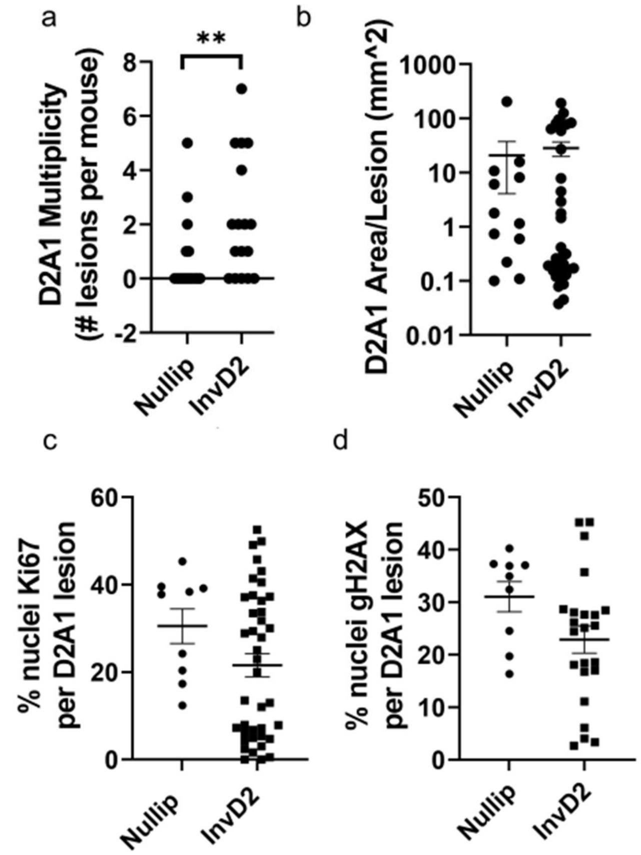

Figure S1. Increased D2A1 metastasis in involution hosts does not associate with enhanced tumor growth. (**a**) Number and (**b**) area of D2A1 metastases per mouse in nullip (n = 27) and InvD2 (n = 17) groups; Two-tailed T test; IHC quantification of percent of tumor nuclei positive for (**c**) Ki67 and (**d**) γH2AX. ** $p < 0.01$. Figure a and b modified from *Cancer Discovery* DOI: 10.1158/2159-8290.CD-16-0822.

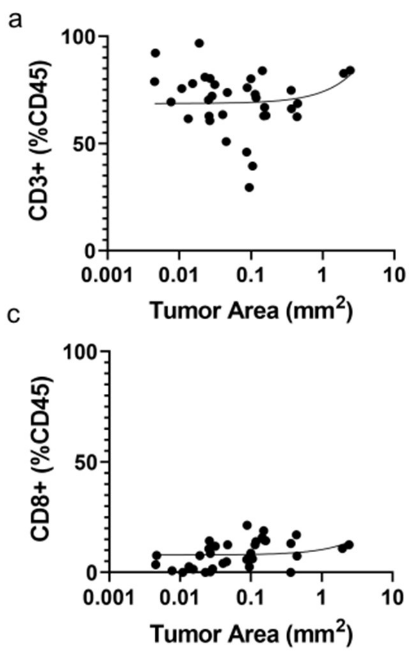

Figure S2. Tumor immune infiltrate does not correlate with tumor size. Pearson's correlation of tumor area (mm^2) with (**a**) CD45+CD3+, (**b**) CD45+CD3+CD4+, and (**c**) CD45+CD3+CD4- (putative CD8+) cells captured by multiplex IHC image cytometry.

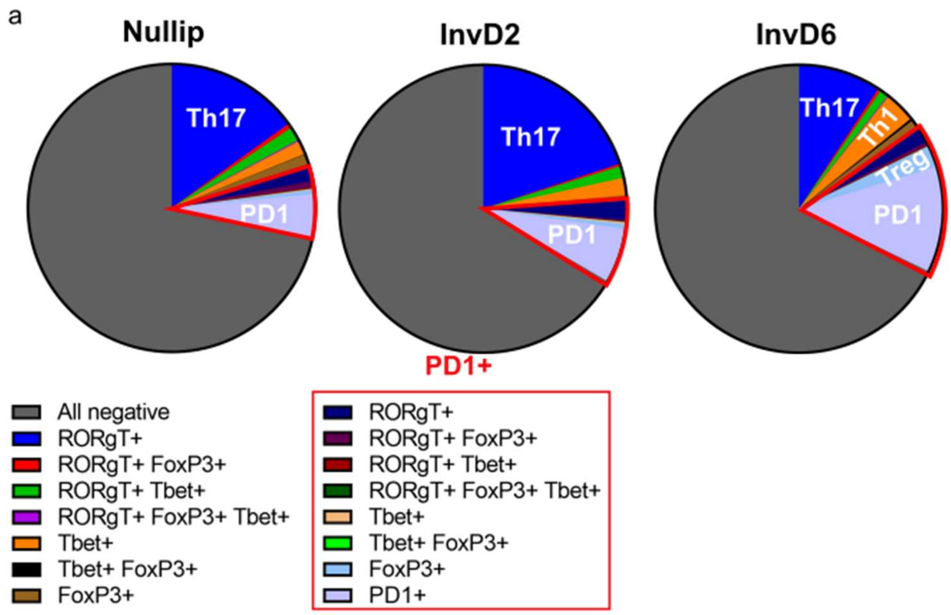

Figure S3. CD4+ T cell polarization is reproductive stage dependent. Flow cytometry phenotyping of CD45+ CD4+ T cells from livers of mice at nullip, InvD2, and InvD6 reproductive stages (nullip n = 8, InvD2 n = 5, InvD6 n = 6). Data represent percent of total CD45+CD4+ T cells.

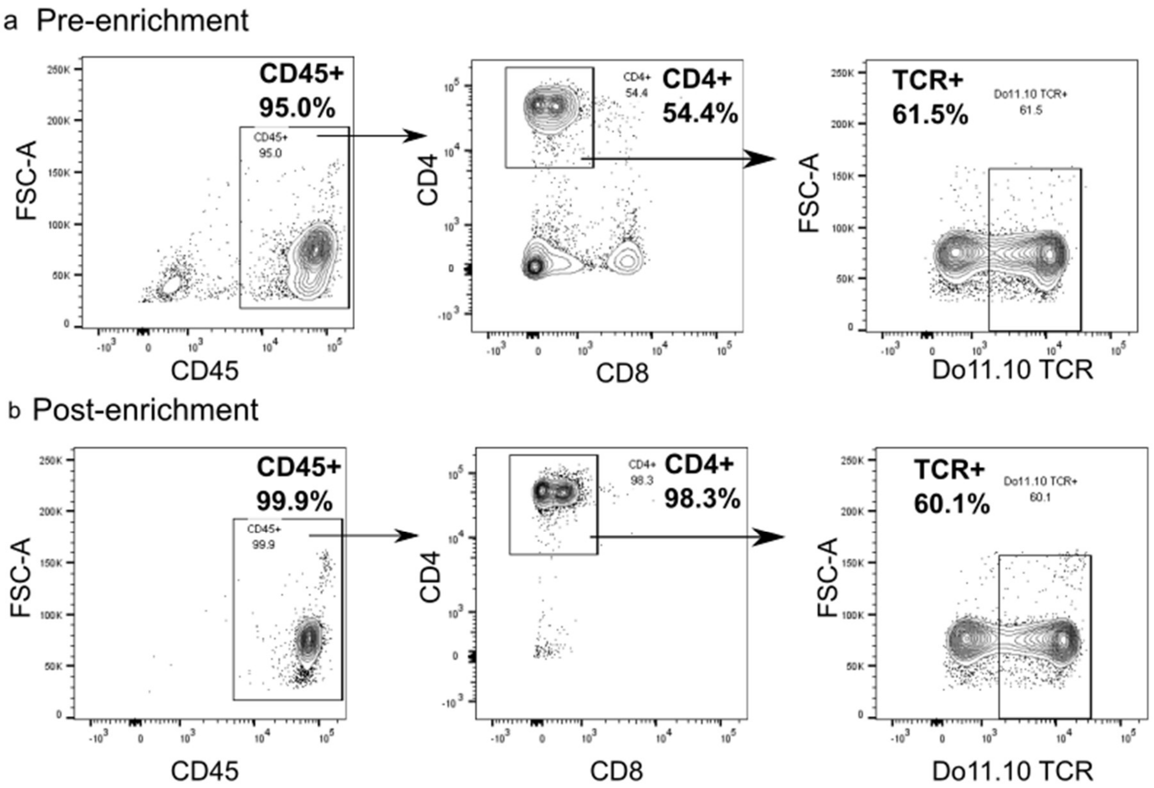

Figure S4. Representative flow cytometry plots demonstrate enrichment of CD4+ cells from whole spleen for adoptive transfer in vivo T cell activation experiment. Flow cytometry plots for CD45+, CD4+, and Do11.10 TCR+ in isolated splenocytes from Do11.10 transgenic mice (**a**) pre- and (**b**) post-enrichment with CD4+ negative selection kit.

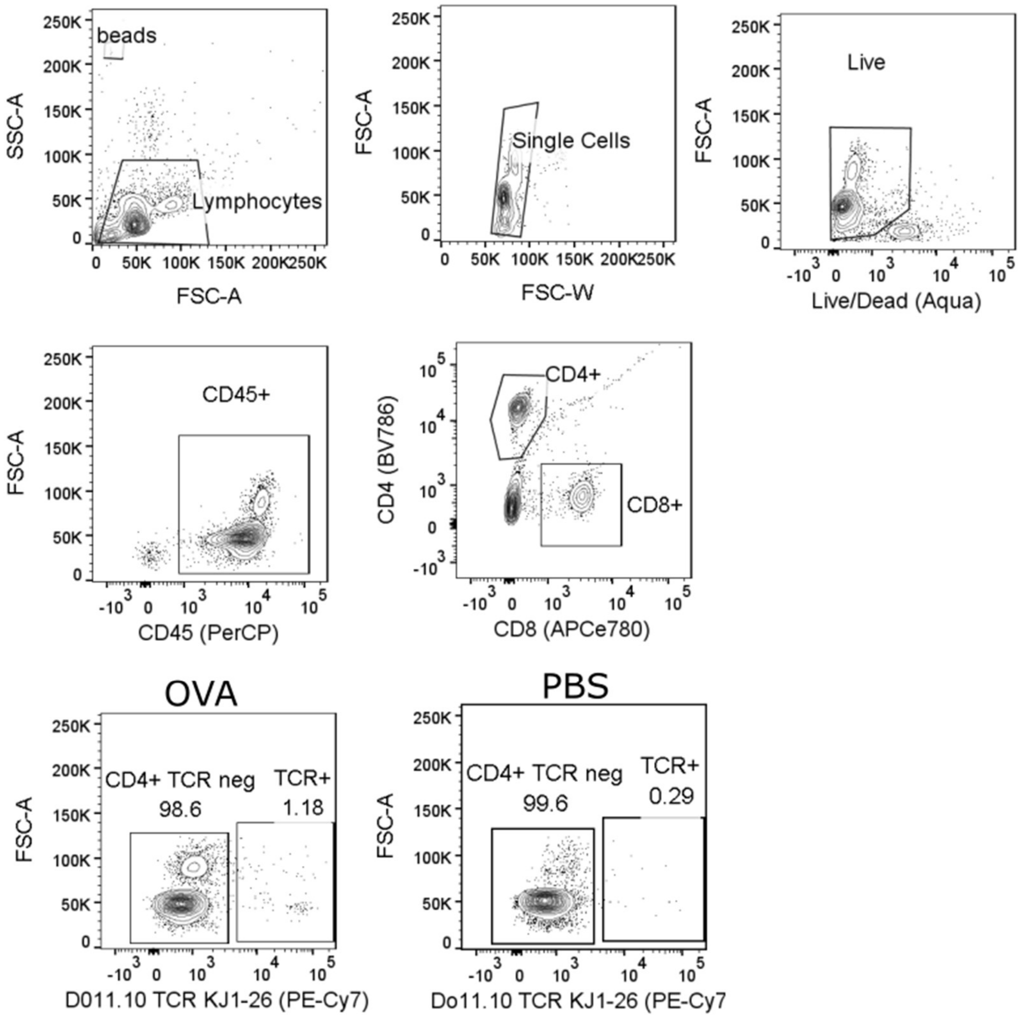

Figure S5. Representative flow cytometry gating for adoptive transfer in vivo T cell activation experiment. Flow cytometry gating scheme for lymphocytes, single cells, live cells, CD45+, CD4+, and CD8+ (upper five panels), and CD4+TCR+/− in the presence of OVA antigen or PBS control (bottom two panels).

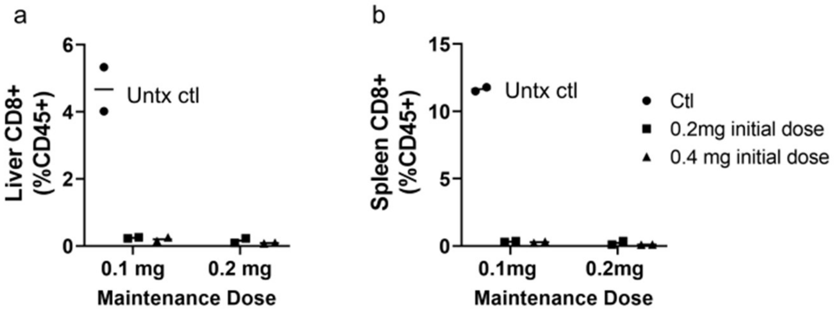

Figure S6. Evidence that lowest initial and maintenance dose concentrations of CD8-depleting antibody effectively depletes CD8+ T cells in the liver and spleen**.** Flow cytometry quantification of CD45+CD3+CD4-CD8b+ T cells in the (**a**) liver and (**b**) spleen with different combinations of CD8-depleting antibody concentrations: initial dose: 0.0 mg, 0.2 mg, 0.4 mg and maintenance dose: 0.0mg, 0.1 mg, 0.2 mg. Circle symbol represents untreated controls (0.0 mg), square symbol represents 0.2 mg initial dose, and triangle symbol represents 0.4mg initial dose. The lower initial (0.2 mg) and lower maintenance (0.1 mg) dose concentrations resulted in same level of CD8+ depletion as higher dose combinations.
